# Optimizing wound healing and cosmesis of surgical closures on rhinophymatous skin

**DOI:** 10.1016/j.jdcr.2024.02.005

**Published:** 2024-02-19

**Authors:** Chiara Rosenbaum, Michael Whitworth

**Affiliations:** Department of Dermatology, Beaumont Health, Trenton, Michigan

**Keywords:** corticosteroids, cosmesis, cutaneous surgery, intralesional triamcinolone acetonide, Mohs micrographic surgery, nasal defects, phymatous rosacea, rhinophyma, wound healing

## Introduction

There is limited literature on rosaceaform tissue and its less-than-optimal healing in the setting of dermatologic surgery. Despite our evolving understanding of the inflammatory pathogenesis of rosacea and its subtypes, there are no clear guidelines for optimizing cosmesis in patients with rosacea who undergo cutaneous surgery.^1^^,^^2^ Phymatous rosacea—which presents with telangiectasias, sebaceous hyperplasia, dilated follicular pores, and increased skin thickening—poses a distinct reconstructive challenge for the dermatologic surgeon.^1^^,^^3^

Rosacea and its subtypes are characterized by dysregulation of the neuroimmune communication network that controls vascular responses.^4^ Rhinophyma is a subtype that is less understood, but it is most likely an advanced rosacea stage with findings of edema, sebaceous hypertrophy, hypervascularity, and fibrosis.^1^ A study evaluating the histopathology of rhinophyma noted that perivascular and periadnexal lymphocytic infiltrate was present in all patients. In addition, mast cells seemed to contribute to the local vasodilation, angiogenesis, and tissue fibrosis observed in tissue samples of patients.^2^

Performing Mohs micrographic surgery (MMS) on phymatous skin can prove challenging given the histologic features associated with the disease. Chronically inflamed and highly sebaceous skin makes for low compliance and difficult surface skin edge union. In addition, excess fibrosis most likely interferes with proper wound approximation and the normal healing process.^5^ As a result, rhinophymatous skin is at increased risk of dehiscence and has a tendency for more visible, beaded incision lines and lumpiness following surgical closure.^6^

**Fig 1:**
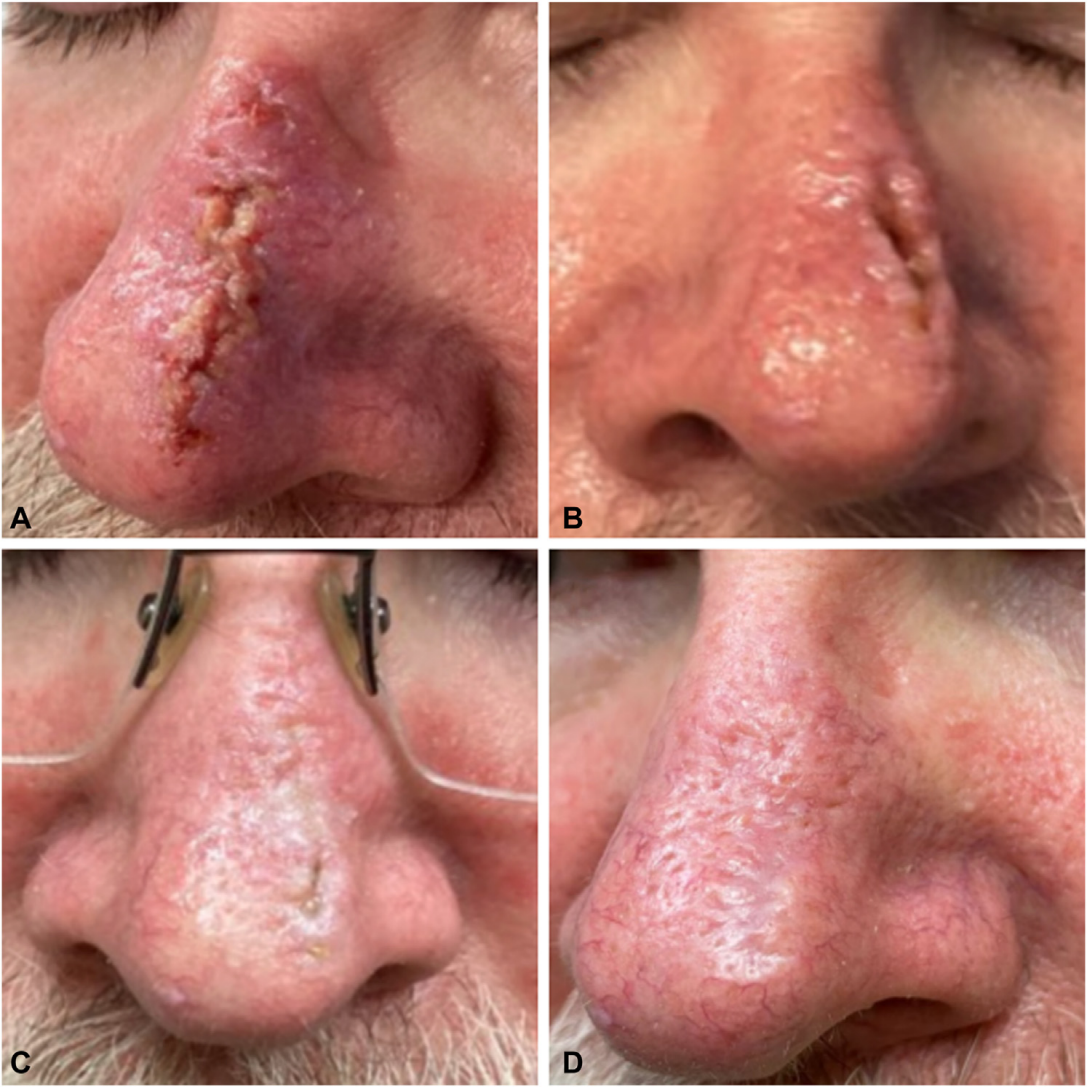
**A,** Initial wound dehiscence 7 days after surgical closure. **B**, Second dehiscence 7 days after previous repair. Patient received intralesional corticosteroid at this visit. **C**, Suture removal 11 days later. **D**, Five months after first initial closure.

This case series was partially inspired by a patient who had a primary closure on the dorsal aspect of the nose after removal of a basal cell carcinoma with MMS. The patient had rhinophymatous skin and experienced wound dehiscence, which was repaired 7 days postprocedure. He was also given a 6-day course of sarecycline 150 mg. Unfortunately, the patient required an additional repair 7 days later, at which point the nasal tissue surrounding the wound was injected with a total of 0.2 mL of triamcinolone acetonide 5 mg/mL. The patient returned 11 days later for suture removal with no evident dehiscence, and the wound was rechecked 1 month and 4 months after (Fig 1). The delayed, but ultimately successful, outcome of wound healing in this patient led the authors to explore how intralesional corticosteroids (CS) may have aided this process and whether it could benefit similar nasal closures on rosacea-prone skin.

We present a series of 4 cases of patients who demonstrated early signs of rhinophyma and underwent MMS for the removal of skin cancer on the nose. They received intralesional triamcinolone immediately after surgical closure with the objective to reduce swelling and decrease inflammation to prevent dehiscence. The findings suggest a potential role for intralesional CS in tissue repair of rhinophymatous skin after cutaneous surgery. All patients here gave consent to the authors and have signed consent forms for their photographs and relevant medical information to be published.

## Case series

Over the course of 9 months, 4 male patients with mild-to-moderate signs of rhinophyma underwent MMS for the removal of skin cancer on their noses at an out-patient dermatology clinic. The rhinophyma severity index is a clinical rhinophyma score that evaluates and stratifies disease by skin thickness.^7^ This score was used to standardize the degree of rhinophyma in our patients. Patient’s age, rhinophyma severity index score, tumor type and location, final defect size, repair type, and final wound area/total repair length were collated and are summarized in Table I. Each defect was undermined at the surgical margins and hemostasis was achieved with electrocautery. The subcutaneous tissue and dermis were closed with 5-0 buried vertical mattress sutures, and 5-0 nylon was used to achieve epidermal closure with either simple interrupted, cross stitches, and/or running sutures.

**Table I:** Patient characteristics and surgical outcomes

Variable	Patient 1	Patient 2	Patient 3	Patient 4
Age	67	83	78	75
RHISI score	2	2	1	1
Tumor type and location	BCC, nasal supratip	BCC, nasal infratip	BCC right nasal ala	BCC nasal infratip
Final defect size	1.2 × 1.1 cm	1.1 × 1.0 cm	0.9 × 0.9 cm	0.8 × 0.7 cm
Repair length	Burow’s advancement flap	Rotation flap	Burow’s advancement flap	Primary closure
Final wound length/total repair area	12.66 cm^2^	6.85 cm^2^	6.51 cm^2^	2 cm

*BCC*, Basal cell carcinoma; *RHISI*, rhinophyma severity index.

**Fig 2:**
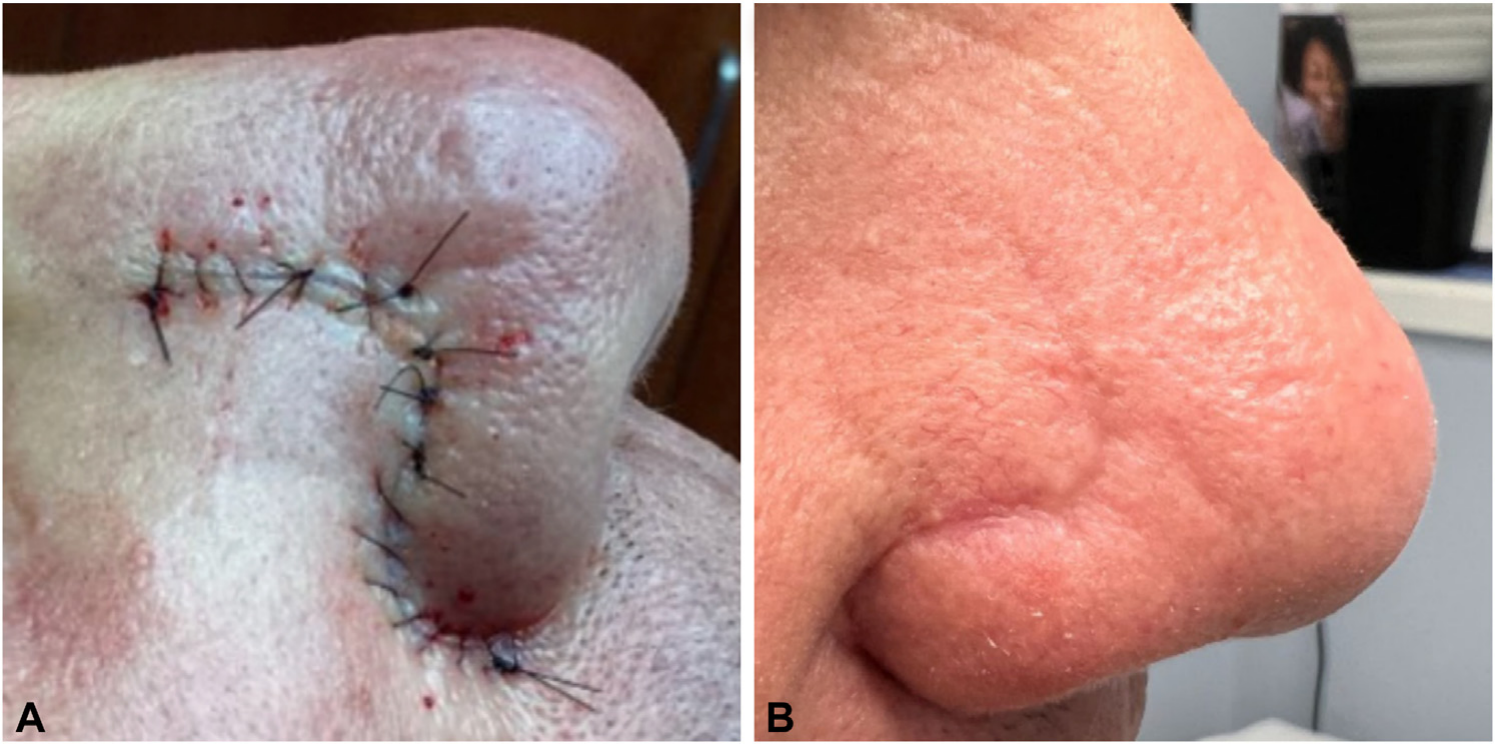
**A,** Patient 3 surgical closure after administration of intralesional triamcinolone acetonide. **B**, Two months later.

**Fig 3:**
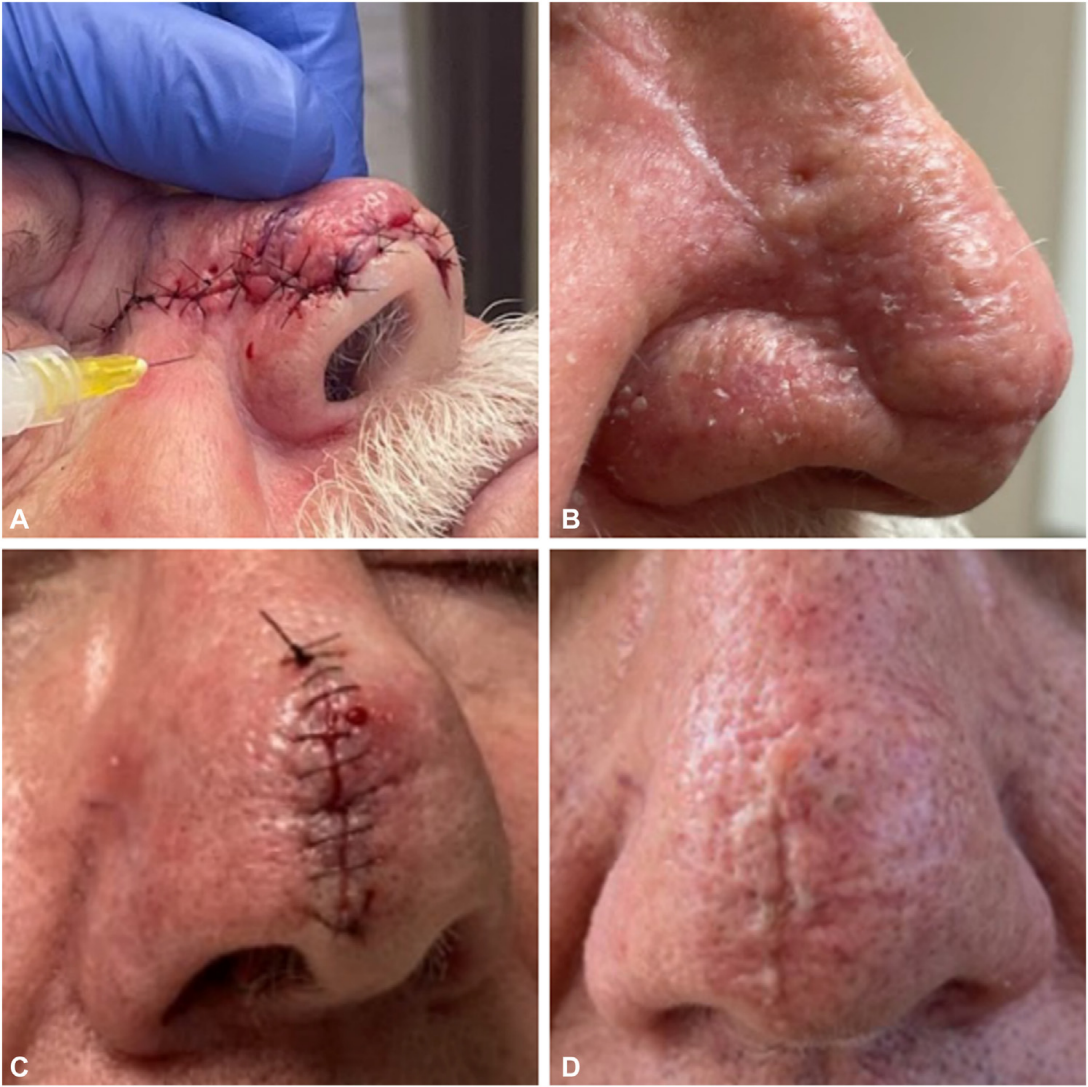
**A,** Patient 1 receiving intralesional corticosteroid immediately after surgical closure of nasal defects. **B**, Patient 1 at 1 month follow-up. **C**, **D**, Patient 4 on day of surgery, and 1 month follow-up.

Immediately after the surgical closure, each patient received injections of 0.2 to 0.5 mL of triamcinolone acetonide 5 mg/mL into the adjacent tissue. There was a total of 4 to 6 injection points administered approximately 5 mm from the sutured wound edge. Clinical response was assessed at suture removal and 1 to 2 months postoperatively. Intervention success was determined by proper wound healing, which we measured by wound edge approximation, lack of track marks, reduced crusting, and smooth skin texture around the surgical site. The main concerns with this intervention were delayed wound healing and atrophy; however, neither were observed in our patients and all cases demonstrated proper tissue repair. The 4 patients in this series experienced proper wound healing with no dehiscence, track marks, or extensive crusting (Fig 2). Three of the patient’s skin texture and wound edge approximation was substandard (Fig 3).

## Discussion

The cutaneous inflammation that characterizes rosacea and its subtypes results from neurovascular hyperreactivity and dysregulation of the innate and adaptive immune system.^4^ This chronic inflammation, along with sebaceous hyperplasia and fibrosis, contribute to the development and progression of rhinophyma.^1^ Common histopathologic findings in rhinophyma include inflammatory lymphocytic infiltrate, high-mast cell burden, telangiectasias, and local angiogenesis.^2^ The unchecked immune response in rhinophyma creates an unfavorable environment for wound healing, and thus, the first phase of wound healing likely compounds this.

In the immediate (24-72 hours) postoperative period, the inflammatory response seems more prominent in patients with rhinophymatous skin, and it is likely that this overactivity hinders wound healing. It is the author’s experience that phymatous skin is more prone to detrimental swelling and that it dehisces more often than surgical closures of normal tissue. Therefore, by decreasing the amount of inflammation immediately after wound closure, injection of local corticosteroid conceivably improves the tissue environment and recalibrates the inflammatory response to a level that is more advantageous for tissue repair. This would explain why our 4 patients did not experience wound dehiscence with the postoperative intervention.

By quieting the innate immune system via cytokine alterations, CS can regulate the aberrant inflammatory response. For instance, CS decrease nuclear factor κ light-chain enhancer of activated B cells, which serves as a transcription factor for proinflammatory cytokines, adhesion molecules, and growth factors.^8^ In addition, CS inhibit both lymphocytic and mast cell activity while also preventing angiogenesis.^9^

Given that rhinophyma often consists of thickened, fibrotic skin, the inhibitory effect of CS on fibroblast cell growth can benefit the immediate postoperative course.^1^ CS counteract fibroblast proliferation by decreasing collagen synthesis, inducing local vasoconstriction, and limiting tissue oxygenation and nutrition.^10^ Although excess corticosteroid results in atrophy and poor wound healing, the moderate dose implemented in this case series most likely aids in improving tissue compliance and remodeling skin texture.

There is a shortage of literature in dermatologic surgery for optimizing cosmetic outcomes in patients with phymatous skin. This case series delineates the potential benefits of intralesional CS for improved postoperative results on rhinophymatous skin. Preventing wound dehiscence is a priority and a prerequisite to superior cosmesis, and CS may aid in normalizing the tissue repair process in this subset of patients. However, the authors also demonstrate that it is still challenging to achieve even skin texture despite such an intervention. Because dermatologic surgeons frequently operate on phymatous sites, such as the nose, it is pertinent to further explore interventions that can improve healing and cosmetic outcomes for patients.

## Conflicts of interest

None disclosed.

## References

[1] Chauhan R., Loewenstein S.N., Hassanein A.H. (2020). Rhinophyma: prevalence, severity, impact and management. Clin Cosmet Investig Dermatol.

[2] Schüürmann M., Wetzig T., Wickenhauser C., Ziepert M., Kreuz M., Ziemer M. (2015). Histopathology of rhinophyma – a clinical-histopathologic correlation. J Cutan Pathol.

[3] Fink C., Lackey J., Grande D.J. (2018). Rhinophyma: a treatment review. Dermatol Surg.

[4] Steinhoff M., Buddenkotte J., Aubert J. (2011). Clinical, cellular, and molecular aspects in the pathophysiology of rosacea. J Investig Dermatol Symp Proc.

[5] Weber P.J., Moody B.R. (2002). Surgical rosacea. Dermatol Surg.

[6] Kwah R.Y., Lawrence C. (2011). Wound management in a patient with rhinophyma and basal cell carcinoma. J Am Acad Dermatol.

[7] Wetzig T., Averbeck M., Simon J.C., Kendler M. (2013). New rhinophyma severity index and mid-term results following shave excision of rhinophyma. Dermatology.

[8] Vandevyver S., Dejager L., Tuckermann J., Libert C. (2013). New insights into the anti-inflammatory mechanisms of glucocorticoids: an emerging role for glucocorticoid-receptor-mediated transactivation. Endocrinology.

[9] Boumpas D.T., Chrousos G.P., Wilder R.L., Cupps T.R., Balow J.E. (1993). Glucocorticoid therapy for immune-mediated diseases: basic and clinical correlates. Ann Intern Med.

[10] Tziotzios C., Profyris C., Sterling J. (2012). Cutaneous scarring: pathophysiology, molecular mechanisms, and scar reduction therapeutics part II. Strategies to reduce scar formation after dermatologic procedures. J Am Acad Dermatol.

